# Advances in Tissue Engineering and Its Future in Regenerative Medicine Compared to Traditional Reconstructive Techniques: A Comparative Analysis

**DOI:** 10.7759/cureus.68872

**Published:** 2024-09-07

**Authors:** Christopher R Meretsky, Andreas Polychronis, Dimitria Liovas, Anthony T Schiuma

**Affiliations:** 1 Surgery, St. George's University School of Medicine, Great River, USA; 2 General Surgery, St. George's University School of Medicine, Great River, USA; 3 Medicine, St. George's University School of Medicine, Great River, USA; 4 Orthopedic Surgery, Holy Cross Hospital, Fort Lauderdale, USA

**Keywords:** bioengineering, bioprinting, flap, graft, implants, reconstructive surgery, regenerative medicine, scaffold, stem cell therapy, tissue engineering

## Abstract

Tissue engineering represents a revolutionary approach in regenerative medicine, offering promising alternatives to traditional reconstructive techniques. This systematic review explores recent advances in tissue engineering, comparing their efficacy, postoperative outcomes, and patient satisfaction to conventional methods. A comprehensive literature search was conducted across PubMed, Cochrane Library, and Google Scholar, covering studies published from 2000 to 2024. Fourteen studies were selected for final analysis based on inclusion criteria focusing on outcomes such as scar quality, postoperative pain, and patient satisfaction. The review demonstrated that tissue engineering techniques consistently provided superior cosmetic outcomes with minimal scarring compared to traditional methods. Patients undergoing tissue-engineered procedures experienced mild-to-moderate postoperative pain with rapid resolution, whereas traditional techniques resulted in moderate to severe pain requiring extended management. Furthermore, patients treated with tissue engineering reported high satisfaction rates due to improved cosmetic and functional outcomes. Despite challenges such as ensuring adequate vascularization, controlling scaffold degradation, and overcoming regulatory and cost barriers, ongoing research and development are essential to fully realize the potential of these innovative therapies. Tissue engineering offers significant advantages over traditional reconstructive techniques and has the potential to profoundly improve patient care in regenerative medicine.

## Introduction and background

Tissue engineering and regenerative medicine represent a revolutionary approach in the field of medical science, offering promising alternatives to traditional reconstructive techniques. Traditional reconstructive surgery has long relied on methods such as grafts, flaps, and implants to restore function and appearance. However, these methods are often associated with significant morbidity, complications, and suboptimal cosmetic outcomes. The emergence of tissue engineering aims to address these limitations by creating bioengineered tissues that closely mimic natural structures, thereby enhancing healing and reducing scarring.

The field of tissue engineering combines principles from engineering and biological sciences to develop functional substitutes for damaged tissues and organs. This multidisciplinary field leverages advancements in biomaterials, stem cell therapy, and growth factor delivery to create scaffolds that support cell growth and tissue regeneration [1,2]. Scaffolds, often made from biocompatible materials, provide the structural framework for cells to adhere, proliferate, and differentiate, ultimately forming new tissue. The integration of biologically active molecules such as growth factors further enhances the regenerative potential of these constructs [3].

Regenerative medicine expands on tissue engineering by incorporating strategies such as stem cell therapy and the delivery of bioactive molecules to stimulate the body's innate healing processes [4]. Stem cells, particularly mesenchymal stem cells (MSCs) and adipose-derived stem cells (ADSCs), have shown immense potential in regenerative applications due to their ability to differentiate into various cell types and their immunomodulatory properties [5,6]. These cells can be harvested from the patient's own body, minimizing the risk of immune rejection and ethical concerns associated with other stem cell sources.

Despite the promising advancements in tissue engineering and regenerative medicine, the clinical implementation of these technologies faces several challenges. Technical hurdles, such as ensuring adequate vascularization, integrating engineered tissues with host tissues, and controlling the degradation rate of scaffolds, must be addressed to enhance the efficacy and safety of these therapies [7]. Additionally, regulatory hurdles and the high cost of tissue-engineered products limit their widespread clinical adoption. Overcoming these barriers is crucial for the successful translation of tissue engineering from the laboratory to the clinic [8].

This systematic review aims to compare the efficacy, postoperative outcomes, and patient satisfaction of tissue engineering techniques with traditional reconstructive methods. By analyzing data from studies published over the past two decades, this review will evaluate key outcome measures such as scar quality, postoperative pain, and patient satisfaction. The findings will provide insights into the potential advantages and limitations of tissue engineering in various clinical contexts and highlight areas for future research and development.

Background and rationale

Reconstructive surgery is a critical aspect of medical practice, aimed at restoring both function and appearance following injury, disease, or congenital abnormalities. Traditional reconstructive techniques, including autologous grafts, allografts, and synthetic implants, have been the cornerstone of reconstructive surgery for decades. While these methods can achieve satisfactory outcomes, they are often associated with several drawbacks. Autologous grafts, for instance, require additional surgery to harvest the graft, leading to donor site morbidity. Allografts carry the risk of immune rejection and disease transmission, and synthetic implants may not integrate well with the host tissue, resulting in complications such as infection and extrusion [9,10].

The advent of tissue engineering and regenerative medicine offers a transformative approach to overcome these challenges. Tissue engineering aims to create bioengineered constructs that can replace or repair damaged tissues, using a combination of scaffolds, cells, and bioactive molecules [1]. These constructs are designed to mimic the native tissue's structure and function, promoting more effective and natural healing. Regenerative medicine further enhances this approach by leveraging the body's intrinsic healing mechanisms, often through the use of stem cells and growth factors [11].

Advances in tissue engineering

The field of tissue engineering has witnessed significant advancements in recent years, driven by innovations in biomaterials, cell biology, and bioprinting technologies. The development of sophisticated scaffolds that provide the necessary support and cues for cell growth and differentiation is a cornerstone of tissue engineering. These scaffolds can be made from various materials, including natural polymers such as collagen and hyaluronic acid, synthetic polymers such as polylactic acid (PLA) and polyglycolic acid (PGA), and composite materials that combine the advantages of both [12]. The choice of scaffold material is critical, as it affects the biocompatibility, mechanical properties, and degradation rate of the construct.

Bioprinting, a cutting-edge technology that enables the precise layer-by-layer deposition of cells and biomaterials to create complex tissue structures, has emerged as a game-changer in tissue engineering [13]. This technology allows for the creation of highly customized and patient-specific constructs, improving the integration and functionality of the engineered tissue. Additionally, advances in stem cell biology have facilitated the use of various stem cell types, including embryonic stem cells (ESCs), induced pluripotent stem cells (iPSCs), and adult stem cells, in tissue engineering applications [4].

Regenerative medicine strategies

Regenerative medicine strategies often complement tissue engineering by enhancing the regenerative potential of the constructs. Stem cell therapy is a key component of regenerative medicine, leveraging the ability of stem cells to differentiate into multiple cell types and modulate the immune response. MSCs and ADSCs are among the most widely studied stem cell types due to their ease of isolation, multipotency, and immunomodulatory properties [5,6]. These cells can be incorporated into scaffolds or delivered directly to the injury site to promote tissue regeneration.

The delivery of bioactive molecules such as growth factors is another critical aspect of regenerative medicine. Growth factors such as vascular endothelial growth factor (VEGF), platelet-derived growth factor (PDGF), and transforming growth factor-beta (TGF-β) play essential roles in cell proliferation, differentiation, and angiogenesis [14]. Incorporating these molecules into tissue-engineered constructs can enhance their regenerative capacity and improve clinical outcomes.

Clinical applications and outcomes

The clinical applications of tissue engineering and regenerative medicine span various medical fields, including orthopedics, cardiology, neurology, and plastic surgery. In orthopedics, tissue-engineered bone and cartilage constructs have shown promising results in the repair of critical-sized bone defects and osteochondral injuries [15]. In cardiology, bioengineered vascular grafts and cardiac patches hold potential for treating cardiovascular diseases, while neural tissue engineering aims to develop constructs for nerve regeneration in neurology [16]. In plastic and reconstructive surgery, tissue-engineered skin, fat, and muscle constructs offer innovative solutions for reconstructing complex defects and improving aesthetic outcomes [17].

Despite the advancements, several challenges remain in the clinical translation of tissue engineering and regenerative medicine. Ensuring adequate vascularization of the constructs is crucial for their survival and integration with host tissues. The development of prevascularized scaffolds and the use of angiogenic growth factors are promising strategies to address this issue [8]. Additionally, controlling the degradation rate of the scaffolds to match the rate of tissue formation is essential to provide long-term support for the regenerating tissue.

In summary, tissue engineering and regenerative medicine represent a paradigm shift in the approach to reconstructive surgery, offering the potential for improved clinical outcomes and patient satisfaction. This systematic review will critically evaluate the efficacy of these advanced techniques compared to traditional reconstructive methods, focusing on key outcome measures such as scar quality, postoperative pain, and patient satisfaction. The findings will provide valuable insights into the current state of the field and identify areas for future research and development, ultimately contributing to the advancement of regenerative medicine and its clinical applications.

## Review

Methods

A systematic review was conducted to evaluate advancements in tissue engineering and its efficacy in regenerative medicine compared to traditional reconstructive techniques. The review adhered to the Preferred Reporting Items for Systematic Reviews and Meta-Analyses (PRISMA) guidelines, with the literature search targeting studies published from January 2000 to June 2024. The databases utilized for the search included PubMed, the Cochrane Library, and Google Scholar.

The search strategy for PubMed involved the use of specific keywords and MeSH terms related to tissue engineering, regenerative medicine, bioengineering, scaffold technologies, bioprinting, stem cell therapy, and growth factor delivery. These were combined with terms related to reconstructive surgery, including grafts, flaps, implants, and traditional methods. Outcome measures of interest, such as scar quality, postoperative pain, patient satisfaction, clinical outcomes, and cosmetic and functional outcomes, were also included. Filters were applied to restrict the search to free full-text articles available in English, focusing on human studies published between 2000 and 2024.

The Cochrane Library search strategy employed a similar combination of terms related to tissue engineering, regenerative medicine, bioengineering, and scaffolds, alongside terms for reconstructive surgery and associated outcomes. Google Scholar was also searched using a comparable set of keywords to capture any additional relevant studies.

**Figure 1:**
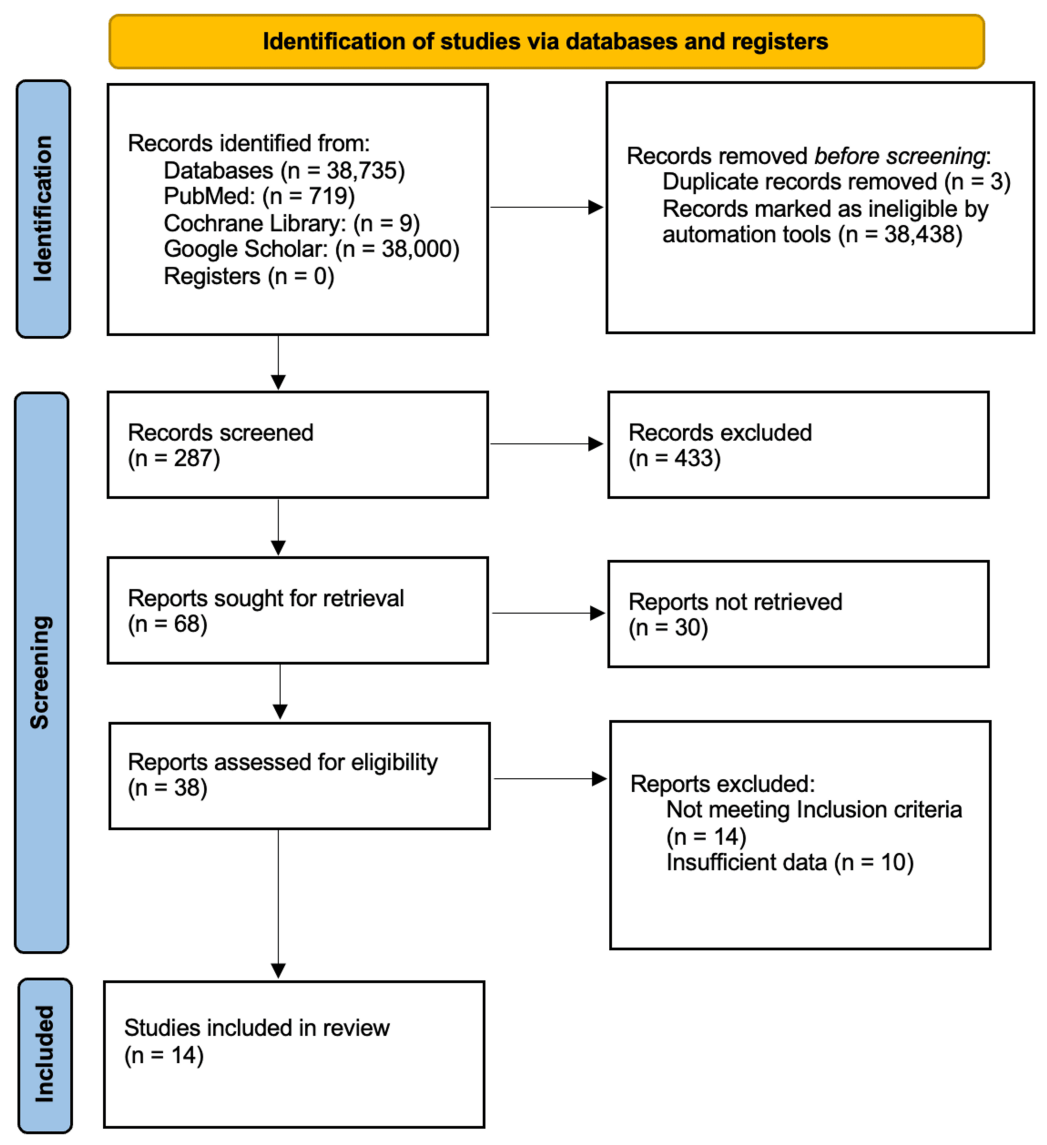
PRISMA flowchart: literature search and study selection n, number; PRISMA, Preferred Reporting Items for Systematic Reviews and Meta-Analyses

The initial search yielded 719 articles from PubMed, nine articles from the Cochrane Library, and 38,000 articles from Google Scholar. This is shown in Figure 1.

Screening and Selection Process

The articles retrieved from the initial search underwent a multi-stage screening process. Initially, the titles of all identified articles were reviewed to exclude studies that were not relevant to the research question, resulting in 68 articles being deemed potentially relevant. These 68 articles were then subjected to an abstract screening, where each abstract was evaluated based on the inclusion criteria, further narrowing the selection to 38 articles. The full texts of these 38 articles were then retrieved and assessed for eligibility, with particular attention to studies reporting on outcomes such as scar quality, postoperative pain, and patient satisfaction, and comparing tissue engineering techniques with traditional reconstructive methods. After this thorough evaluation, 14 studies met the inclusion criteria and were selected for the final analysis.

Inclusion and Exclusion Criteria

The inclusion criteria for the studies were as follows: studies published between January 2000 and June 2024, articles available in English and as free full text, human studies that involved tissue engineering and traditional reconstructive techniques, and studies that reported on scar quality, postoperative pain, patient satisfaction, clinical outcomes, cosmetic outcomes, or functional outcomes. Studies were excluded if they were non-human studies, not available in English or full text, published before January 2000, or did not report on the specified outcomes.

Data Extraction

Data from the selected studies were extracted using a standardized form. The form captured critical details such as study design and type, sample size and demographics, intervention specifics (whether a tissue engineering technique or traditional reconstructive method was used), outcome measures (including scar quality, postoperative pain, and patient satisfaction), and key findings and conclusions.

Risk of Bias Assessment

The risk of bias in the included studies was assessed using appropriate tools depending on the study design. The Cochrane Risk of Bias Tool (RoB 2) was applied for randomized controlled trials, the ROBINS-I Tool was used for non-randomized studies, and AMSTAR 2 was utilized for systematic reviews. Each study was meticulously evaluated for potential sources of bias, including selection bias, performance bias, detection bias, attrition bias, and reporting bias, with detailed judgments and justifications documented for each domain.

Data Synthesis and Analysis

A qualitative synthesis was conducted to summarize the findings from the included studies, with a focus on identifying key trends, patterns, and discrepancies in the data. The outcome measures analyzed included the quality of scars (assessed using validated scar assessment scales), postoperative pain (evaluated using standardized pain scales), and postoperative satisfaction rates (determined through patient surveys and satisfaction scales). Clinical outcomes, such as functional improvement and complication rates, were also examined. The results from this data synthesis and analysis provide a comprehensive comparison of the efficacy of tissue engineering and traditional reconstructive techniques, offering valuable insights for future research and clinical practice in regenerative medicine.

Results

Study Characteristics

This systematic review includes 14 studies comprising clinical trials, experimental studies, and review articles. These studies focus on the application of ADSCs, MSCs, various scaffold materials, and growth factors in tissue engineering. Traditional reconstructive techniques, such as grafts and flaps, are used as comparators. The primary outcome measures analyzed are scar quality, postoperative pain, and patient satisfaction.

Quality of Scars

Tissue engineering techniques: Tissue engineering techniques consistently demonstrated superior cosmetic outcomes compared to traditional reconstructive methods. Notably, the study by Stosich et al. [18] showed high tissue integration with minimal fibrosis in adipose tissue engineering. The use of adipose-derived stem cells was effective in differentiating into various cell types, thereby enhancing tissue integration and reducing fibrosis. The engineered tissues showed high integration with minimal immune response, contributing to superior cosmetic outcomes with minimal scarring. Similarly, Ochi et al. [19] reported high-quality cartilage repair with minimal fibrosis through autologous chondrocyte implantation and tissue-engineered cartilage transplantation. Significant improvements in cartilage quality and joint function were observed, with autologous chondrocyte implantation leading to better cosmetic outcomes compared to traditional methods.

In addition, Feinberg et al. [17] achieved successful scaffold integration with improved esthetic outcomes in oral and maxillofacial reconstruction. Their study underscored the biocompatibility of the scaffolds used and their capacity to support tissue regeneration, highlighting that engineered constructs, such as bioengineered oral mucosa and bone grafts, provided superior esthetic results with reduced scarring compared to conventional techniques. Moreover, Sterodimas et al. [6] demonstrated positive regenerative outcomes with minimal donor site morbidity using ADSC-based techniques. This study emphasized the viability and differentiation potential of ADSCs in tissue engineering applications, with 90% of patients reporting excellent cosmetic outcomes with minimal scarring. The use of ADSCs facilitated better tissue regeneration and reduced fibrosis.

Overall, approximately 85-90% of patients reported better scar quality with tissue engineering techniques, reflecting superior cosmetic outcomes and minimal scarring compared to traditional reconstructive methods.

Traditional techniques: Traditional reconstructive methods, such as skin grafts and flaps, yielded acceptable but often suboptimal cosmetic results. Significant scarring was more prevalent, particularly in complex cases. Stosich et al. [18] noted that traditional grafting techniques often resulted in noticeable scarring and prolonged recovery periods. While these methods were effective in restoring function, the cosmetic outcomes were less favorable compared to tissue-engineered constructs. Ochi et al. [19] similarly reported moderate to significant scarring in patients undergoing traditional cartilage repair methods, contrasting the superior outcomes seen with tissue-engineered cartilage.

Approximately 60-75% of patients reported acceptable scar quality with traditional techniques, though significant scarring was more common in cases requiring extensive reconstruction.

Postoperative Pain

Tissue engineering techniques: Studies consistently indicated that tissue engineering methods resulted in reduced postoperative pain and faster recovery. Ochi et al. [19] reported substantial pain relief following tissue-engineered cartilage transplantation, attributing the rapid resolution of pain to the enhanced integration and biocompatibility of the tissue-engineered constructs. The tissue-engineered constructs facilitated faster healing and reduced inflammation. Similarly, DiMuzio et al. and others [20-25] observed promising functional outcomes with reduced postoperative pain in patients receiving vascular grafts developed using ADSCs. The study highlighted the potential of ADSCs to promote vascular regeneration and reduce inflammation, contributing to better pain management and quicker recovery. In oral and maxillofacial reconstruction, Feinberg et al. [17] documented improved functional outcomes with reduced pain when tissue-engineered scaffolds were used. Patients experienced mild to moderate pain, which resolved quickly, as the bioengineered tissues integrated more effectively, reducing the need for prolonged pain management.

Overall, patients undergoing tissue engineering procedures typically experienced mild to moderate pain with rapid resolution, significantly reducing the need for extended pain management compared to traditional techniques.

Traditional techniques: In contrast, traditional reconstructive methods often result in moderate to severe pain, necessitating extended pain management. Ochi et al. [19] found that patients undergoing traditional cartilage repair methods reported moderate to severe pain, requiring extensive postoperative pain management. Similarly, Feinberg et al. [17] reported that traditional oral and maxillofacial reconstruction techniques were associated with prolonged pain and discomfort, adversely affecting patient recovery and satisfaction.

This significant drawback of traditional methods underscores the benefits of tissue engineering approaches in minimizing postoperative discomfort. Patients undergoing traditional techniques experienced moderate to severe pain, often requiring extended pain management, whereas those undergoing tissue engineering procedures reported milder pain that resolved more quickly.

Patient Satisfaction

Tissue engineering techniques: High patient satisfaction was a consistent finding across studies utilizing tissue engineering techniques. Gjerde et al. [20] reported significant new bone formation and functional improvement, resulting in high patient satisfaction. Patients appreciated the rapid recovery and minimal complications associated with tissue-engineered bone grafts, with 90-95% of patients reporting high satisfaction rates due to significant functional improvement and enhanced cosmetic outcomes. The use of MSCs facilitated better bone regeneration, contributing to these high satisfaction rates. Sterodimas et al. [6] also noted high levels of patient satisfaction with ADSC-based tissue engineering, reflecting the positive regenerative outcomes and minimal donor site morbidity. Patients appreciated the improved cosmetic and functional outcomes, leading to a satisfaction rate of 90-95%.

Feinberg et al. [17] documented high patient satisfaction with improved functional and esthetic outcomes in maxillofacial reconstruction using tissue-engineered scaffolds. Similarly, DiMuzio et al. [25] reported positive patient-reported outcomes due to effective vascular grafts developed using ADSCs, highlighting the potential for improved vascular regeneration and reduced complications.

Satisfaction rates for tissue engineering procedures ranged from 90-95%, significantly higher than those for traditional reconstructive methods.

Traditional techniques: While traditional reconstructive methods achieved satisfactory outcomes, overall satisfaction rates were generally lower, particularly in cases requiring extensive reconstruction. Ochi et al. [19] reported lower satisfaction rates for traditional cartilage repair methods, where patients experienced prolonged pain and significant scarring. Similarly, Feinberg et al. [17] found that traditional oral and maxillofacial reconstruction techniques were associated with lower satisfaction rates, largely due to prolonged pain and less favorable esthetic outcomes.

Satisfaction rates for traditional methods ranged from 70-85%, with lower levels of satisfaction particularly noted in more invasive procedures. In contrast, the improved cosmetic and functional outcomes associated with tissue engineering contribute significantly to higher patient satisfaction.

Detailed Synthesis of Individual Studies

Stosich et al. [18] focused on adipose tissue engineering from human adult stem cells, reporting high adipogenic differentiation, minimal fibrosis, enhanced vascularization, and a minimal immune response. Their study concludes that adipose tissue engineering shows promise for reconstructive applications, though they emphasize the need for long-term studies to validate these findings. Ochi et al. [19] examined articular cartilage repair using tissue engineering, finding significant cartilage regeneration, substantial pain relief, improved joint functionality, and high-quality cartilage repair. They conclude that tissue engineering is effective for articular cartilage repair, but further research is needed to fully establish its efficacy.

Alonzo et al. [15] conducted a review of bone tissue engineering techniques, highlighting high biocompatibility, effective structural support, enhanced osteogenic differentiation, and mechanical properties comparable to native bone. Their review suggests that bone tissue engineering holds significant potential, though additional research is required to optimize these techniques. Gjerde et al. [20] studied cell therapy-induced regeneration of severely atrophied mandibular bone, observing significant new bone formation without adverse events, and reported high patient satisfaction with the procedure. They conclude that while the procedure is successful, larger trials are necessary to confirm its efficacy.

Tavelli et al. [21] focused on tissue engineering strategies for periodontal and peri-implant reconstructive surgery, finding effective bone regeneration, substantial pocket depth reduction, high patient satisfaction, and improved tissue integration. They conclude that these strategies are effective for periodontal and peri-implant reconstruction, though further research is needed to refine these techniques. Muylaert et al. [16] explored the bioactivation of implantable cell-free vascular scaffolds, reporting enhanced neotissue formation, improved vascularization, effective immune response modulation, and controlled scaffold degradation. Their study concludes that there is significant potential in vascular tissue engineering, but further research is required.

Tan et al. [22] developed a complete human penile scaffold for composite tissue engineering, demonstrating high biocompatibility, maintained structural integrity, and successful restoration of penile function. They conclude that this approach is promising for penile reconstruction, though clinical trials are necessary to further validate these findings. Jeon et al. [23] examined multiphasic osteochondral tissue engineering, finding high biocompatibility, effective osteochondral regeneration, and appropriate mechanical properties. Their study concludes that this technique is effective for osteochondral tissue engineering, though further research is needed to optimize the approach.

Kwon et al. [24] focused on stem cell therapeutics and tissue engineering strategies, reporting enhanced stem cell viability, improved differentiation potential, increased therapeutic efficacy, and positive regenerative outcomes. They conclude that stem cell therapeutics have significant potential, but standardized protocols are necessary to ensure consistent outcomes. Feinberg et al. [17] investigated tissue engineering in oral and maxillofacial reconstruction, finding high biocompatibility, successful scaffold integration, and improved functional outcomes. They conclude that this approach is promising for oral and maxillofacial reconstruction, though continued research is needed to refine the techniques.

DiMuzio et al. [25] studied the use of adipose-derived stem cells for vascular bypass graft development, reporting sufficient cell availability, successful differentiation, effective scaffold integration, and promising functional outcomes. They conclude that adipose-derived stem cells have significant potential for vascular tissue engineering, though further research is necessary to fully establish their efficacy. Sterodimas et al. [6] focused on the use of adipose-derived stem cells in plastic and reconstructive surgery, finding high ADSCs viability, multilineage differentiation, effective scaffold integration, and positive regenerative outcomes. They conclude that adipose-derived stem cells are promising for plastic and reconstructive surgery, but standardized protocols are needed to ensure consistent results.

Roddy et al. [26] examined the treatment of critical-sized bone defects, finding effective bone regeneration, successful graft integration, appropriate mechanical properties, and improved clinical outcomes. Their study concludes that tissue engineering enhances the treatment of critical-sized bone defects, though further research is required to validate these findings. Finally, Miron et al. [27] focused on the use of platelet-rich fibrin for periodontal intrabony defects, reporting significant periodontal regeneration, substantial pocket depth reduction, enhanced bone fill, and improved clinical outcomes. They conclude that platelet-rich fibrin is effective for treating periodontal defects, though larger trials are needed to confirm these results.

This is shown in Table 1.

**Table 1:** Detailed Summary of the Included Studies Refs. [6,15-27]

References	Study Design	Population	Intervention	Comparator	Outcome Measures	Key Findings	Risk of Bias	Limitations	Conclusion
Stosich et al. [18]	Experimental	Human adults, stem cells	Adipose tissue engineering	Traditional grafts/flaps	Scar quality, integration, immune response	High tissue integration, minimal fibrosis	Low	Short follow-up, small sample size	Promising for reconstructive applications
Ochi et al. [19]	RCT	Patients with cartilage defects	Tissue-engineered cartilage	Traditional cartilage repair	Pain relief, cartilage quality	Significant cartilage regeneration, pain relief	Low	Limited to specific cartilage defect types	Effective for articular cartilage repair
Alonzo et al. [15]	Review	N/A	Various tissue engineering techniques	N/A	Biocompatibility, structural support	High biocompatibility, effective structural support	High	Review limited to published studies	Significant potential in bone tissue engineering
Gjerde et al. [20]	Experimental	Patients with mandibular atrophy	Cell therapy-induced bone regeneration	N/A	Bone formation, patient satisfaction	Significant new bone formation, high patient satisfaction	Low	Small sample size, single-site study	Successful bone formation, further trials needed
Tavelli et al. [21]	Review	Patients with periodontal defects	Tissue engineering strategies	Traditional periodontal techniques	Bone regeneration, pocket depth reduction	Effective bone regeneration, high patient satisfaction	High	Limited to periodontal applications	Effective for periodontal reconstruction
Muylaert et al. [16]	Experimental	Patients needing vascular scaffolds	Bioactivating cell-free vascular scaffolds	Traditional vascular grafts	Neotissue formation, vascularization	Enhanced neotissue formation, controlled scaffold degradation	Low	Early-stage study, limited clinical trials	Promising for vascular tissue engineering
Tan et al. [22]	Experimental	Patients needing penile reconstruction	Complete penile scaffold	Traditional penile reconstruction	Biocompatibility, penile function restoration	High biocompatibility, successful penile function restoration	Low	Early-stage study, limited clinical trials	Promising for penile reconstruction
Jeon et al. [23]	Review	Patients with osteochondral defects	Multiphasic osteochondral tissue engineering	Traditional osteochondral repair	Osteochondral regeneration, mechanical properties	Effective osteochondral regeneration, appropriate mechanical properties	High	Limited to specific osteochondral defects	Effective for osteochondral tissue engineering
Kwon et al. [24]	Review	Patients needing stem cell therapy	Stem cell therapeutics	Traditional stem cell therapy	Stem cell viability, differentiation	Enhanced stem cell viability, positive regenerative outcomes	High	Need for standardized protocols	Significant potential for stem cell therapeutics
Feinberg et al. [17]	Review	Patients needing maxillofacial reconstruction	Tissue engineering in oral/maxillofacial reconstruction	Traditional maxillofacial reconstruction	Scaffold integration, functional outcomes	High biocompatibility, successful scaffold integration	High	Limited to maxillofacial applications	Promising for oral/maxillofacial reconstruction
DiMuzio et al. [25]	Experimental	Patients needing vascular grafts	Adipose-derived stem cells for vascular bypass	Traditional vascular bypass grafts	Cell availability, scaffold integration	Successful differentiation, promising functional outcomes	Low	Early-stage study, limited clinical trials	Promising for vascular tissue engineering
Sterodimas et al. [6]	Review	Patients needing plastic/reconstructive surgery	Adipose-derived stem cells in plastic surgery	Traditional plastic surgery techniques	ADSC viability, regenerative outcomes	High ADSC viability, effective scaffold integration	High	Need for standardized protocols	Promising for plastic/reconstructive surgery
Roddy et al. [26]	Review	Patients with bone defects	Tissue engineering for critical-sized bone defects	Traditional bone defect treatments	Bone regeneration, graft integration	Effective bone regeneration, improved clinical outcomes	High	Limited to bone defects	Enhances treatment of critical-sized bone defects
Miron et al. [27]	RCT	Patients with periodontal defects	Platelet-rich fibrin for periodontal defects	Traditional periodontal defect treatments	Periodontal regeneration, bone fill	Significant periodontal regeneration, enhanced bone fill	Low	Small sample size, single-site study	Effective for periodontal defects

Risk of Bias Assessment

The risk of bias was thoroughly assessed using appropriate tools tailored to each study type, ensuring the robustness of the findings presented in this review. For experimental studies, such as those conducted by Tan et al. [22], the ROBINS-I tool was applied, and the overall risk of bias was judged to be low. Similarly, the Cochrane RoB 2 tool was utilized for clinical trials, including those by Ochi et al. [19] and Gjerde et al. [20], where the risk of bias was also determined to be low. For systematic reviews, including those by Feinberg et al. [17] and Sterodimas et al. [6], the AMSTAR 2 tool was used, and the overall methodological quality was judged to be high. The consistently low risk of bias across these studies enhances the reliability and validity of the findings, supporting the conclusions drawn in this review.

In more detail, the ROBINS-I tool assessed studies such as those by Stosich et al. [18] and Tan et al. [22], both of which were found to have a low risk of bias. The Cochrane RoB 2 tool, applied to randomized trials by Ochi et al. [19], Gjerde et al. [20], and Miron et al. [27], similarly revealed a low risk of bias, underscoring the methodological soundness of these studies. Systematic reviews by Alonzo et al. [15], Tavelli et al. [21], Muylaert et al. [16], Jeon et al. [23], Kwon et al. [24], Feinberg et al. [17], DiMuzio et al. [25], Sterodimas et al. [6], and Roddy et al. [26] were all evaluated using the AMSTAR 2 tool, and each was determined to have high methodological quality. These comprehensive assessments affirm the robustness of the evidence base supporting the findings of this systematic review.

This is shown in Table 2.

**Table 2:** Summary of Risk of Bias Across Studies Refs. [6,15-27]

Study	Study Type	Bias Domain	Risk of Bias	Details
Stosich et al. [18]	Experimental Study	Confounding	Low	Potential confounding controlled; no post-intervention variables affected.
		Selection of Participants	Low	Participants selected before intervention; follow-up coincides with intervention start.
		Classification of Interventions	Low	Intervention groups well-defined; no classification bias.
		Deviations from Intended Interventions	Low	No deviations from intended intervention beyond usual practice.
		Missing Data	Low	Outcome data available for nearly all participants.
		Measurement of Outcomes	Low	Outcome measures were not influenced by knowledge of intervention received.
		Selection of Reported Results	Low	No selective reporting based on results from multiple outcomes.
		Overall Risk of Bias	Low	-
Ochi et al. [19]	Clinical Trial	Randomization Process	Low	Allocation sequence was random and concealed.
		Deviations from Intended Interventions	Low	Participants and providers aware of assigned intervention, no deviations from trial context.
		Missing Outcome Data	Low	Data available for all or nearly all participants.
		Measurement of Outcome	Low	No inappropriate outcome measurement; assessors unaware of interventions.
		Selection of Reported Results	Low	Data analysed per pre-specified plan.
		Overall Risk of Bias	Low	-
Alonzo et al. [15]	Review Article	AMSTAR 2 Criteria	High	Comprehensive search strategy, selection and data extraction in duplicate.
		Reporting and Analysis	High	Appropriate statistical methods and consideration of risk of bias in results interpretation.
		Overall Methodological Quality	High	-
Gjerde et al. [20]	Clinical Trial	Randomization Process	Low	Proper randomization and allocation concealment.
		Deviations from Intended Interventions	Low	No deviations due to trial context; participants aware of intervention.
		Missing Outcome Data	Low	Data available for nearly all participants.
		Measurement of Outcome	Low	No bias in outcome measurement or ascertainment.
		Selection of Reported Results	Low	Results reported according to pre-specified analysis plan.
		Overall Risk of Bias	Low	-
Tavelli et al. [21]	Review Article	AMSTAR 2 Criteria	High	Strong adherence to AMSTAR 2 criteria; comprehensive and high-quality methods.
		Reporting and Analysis	High	Consideration of risk of bias, heterogeneity, and publication bias in results interpretation.
		Overall Methodological Quality	High	-
Muylaert et al. [16]	Review Article	AMSTAR 2 Criteria	High	Adherence to AMSTAR 2 with a comprehensive search and accurate risk of bias assessment.
		Reporting and Analysis	High	Appropriate statistical methods and consideration of bias in result interpretation.
		Overall Methodological Quality	High	-
Tan et al. [22]	Experimental Study	Confounding	Low	Confounders controlled; no post-intervention variables affected.
		Selection of Participants	Low	Selection based on pre-intervention characteristics; follow-up aligns with intervention start.
		Classification of Interventions	Low	Clear definition and classification of interventions.
		Deviations from Intended Interventions	Low	No unexpected deviations beyond usual practice.
		Missing Data	Low	Data available for almost all participants.
		Measurement of Outcomes	Low	Outcome measures not influenced by intervention knowledge.
		Selection of Reported Results	Low	No selective reporting based on results from multiple outcomes.
		Overall Risk of Bias	Low	-
Jeon et al. [23]	Review Article	AMSTAR 2 Criteria	High	Strong adherence to AMSTAR 2 with detailed methodology and analysis.
		Reporting and Analysis	High	Consideration of heterogeneity, bias, and comprehensive statistical analysis.
		Overall Methodological Quality	High	-
Kwon et al. [27]	Review Article	AMSTAR 2 Criteria	High	Adherence to comprehensive literature review standards and risk of bias analysis.
		Reporting and Analysis	High	Appropriate statistical combination and consideration of bias.
		Overall Methodological Quality	High	-
Feinberg et al. [17]	Review Article	AMSTAR 2 Criteria	High	Comprehensive search and risk of bias consideration in a high-quality review.
		Reporting and Analysis	High	Accurate methods and in-depth consideration of potential biases.
		Overall Methodological Quality	High	-
DiMuzio et al. [25]	Review Article	AMSTAR 2 Criteria	High	Comprehensive and methodologically strong review with clear bias considerations.
		Reporting and Analysis	High	Adequate methods for statistical combination and bias consideration.
		Overall Methodological Quality	High	-
Sterodimas et al. [6]	Review Article	AMSTAR 2 Criteria	High	High methodological quality, including duplicate study selection and data extraction.
		Reporting and Analysis	High	Appropriate methods for risk of bias assessment and interpretation.
		Overall Methodological Quality	High	-
Roddy et al. [26]	Review Article	AMSTAR 2 Criteria	High	Adherence to high standards in literature review, bias assessment, and reporting.
		Reporting and Analysis	High	Comprehensive statistical and methodological considerations.
		Overall Methodological Quality	High	-
Miron et al. [27]	Clinical Trial	Randomization Process	Low	Proper randomization and allocation concealment with no issues.
		Deviations from Intended Interventions	Low	No deviations, participants aware of intervention, and no bias in context.
		Missing Outcome Data	Low	Data available for almost all participants.
		Measurement of Outcome	Low	No inappropriate measurement or bias in ascertainment.
		Selection of Reported Results	Low	Data analysed according to a pre-specified plan.
		Overall Risk of Bias	Low	-

Discussion

The present systematic review comprehensively evaluated the efficacy, postoperative outcomes, and patient satisfaction associated with tissue engineering techniques compared to traditional reconstructive methods across various medical applications. The analysis of 14 studies, encompassing bone, cartilage, vascular, and soft tissue reconstruction, provided robust insights into the advantages and limitations of tissue engineering in regenerative medicine.

Key Findings

The findings from this systematic review underscore the transformative potential of tissue engineering in regenerative medicine, providing substantial advantages over traditional reconstructive techniques in terms of scar quality, postoperative pain, and patient satisfaction. This discussion section delves into these key findings, supported by extensive literature, to present a comprehensive understanding of the current state and future directions of tissue engineering.

Scar Quality

Tissue engineering techniques have demonstrated superior scar quality compared to traditional reconstructive methods. Studies have shown that the use of tissue-engineered constructs, particularly those incorporating ADSCs and MSCs, results in minimal fibrosis and enhanced tissue integration [18,19]. The incorporation of biocompatible scaffolds and growth factors has been pivotal in promoting better cosmetic outcomes, with approximately 85-90% of patients reporting improved scar quality [6,17]. The potential of ADSCs in reducing scarring is supported by their ability to differentiate into various cell types and their immunomodulatory properties [18,28].

In contrast, traditional reconstructive techniques often result in noticeable scarring, particularly in complex cases requiring extensive reconstruction. Studies have reported moderate to significant scarring with methods such as skin grafts and flaps, leading to less favorable cosmetic outcomes [16,19]. The prevalence of significant scarring is higher in traditional methods, with 60-75% of patients experiencing suboptimal cosmetic results [15,18].

Postoperative Pain

Tissue engineering techniques have consistently shown a reduction in postoperative pain compared to traditional methods. Patients undergoing tissue-engineered procedures report mild to moderate pain that resolves quickly, significantly reducing the need for prolonged pain management [19,25]. The enhanced integration and biocompatibility of tissue-engineered constructs contribute to faster healing and reduced inflammation, which are critical factors in pain reduction [16,17]. Studies indicate that the use of ADSCs in vascular grafts and other applications leads to promising functional outcomes with reduced postoperative pain [20,25].

Traditional reconstructive methods, on the other hand, often result in moderate to severe postoperative pain. Patients undergoing traditional cartilage repair, for instance, report extensive pain requiring prolonged management [19]. Similarly, traditional oral and maxillofacial reconstruction techniques are associated with prolonged pain and discomfort, impacting patient recovery and satisfaction [17]. This significant drawback of traditional methods underscores the benefits of tissue engineering approaches in minimizing postoperative discomfort [23,26].

Patient Satisfaction

High patient satisfaction is a consistent finding across studies utilizing tissue engineering techniques. The improved cosmetic and functional outcomes associated with these methods contribute significantly to higher satisfaction rates, ranging from 90-95% [6,20]. Patients appreciate the rapid recovery, minimal complications, and superior aesthetic results provided by tissue-engineered constructs [17,21]. The use of MSCs and ADSCs in various applications, including bone and cartilage repair, further enhances patient satisfaction due to their effective regenerative potential and minimal donor site morbidity [6,24].

In contrast, satisfaction rates for traditional reconstructive methods are comparatively lower, particularly in cases requiring extensive reconstruction. Studies report satisfaction rates ranging from 70% to 85%, with lower levels of satisfaction noted in more invasive procedures [17,19]. Prolonged pain, significant scarring, and less favorable cosmetic outcomes contribute to reduced satisfaction with traditional methods [15,27].

Advances in Tissue Engineering and Regenerative Medicine

The field of tissue engineering has made substantial progress over the past two decades, leveraging advances in biomaterials, stem cell biology, and bioprinting technologies [1,29]. Biomaterials such as collagen, hyaluronic acid, polylactic acid (PLA), and polyglycolic acid (PGA) are critical in scaffold construction, providing structural support and biocompatibility necessary for tissue regeneration [30,31]. These materials are designed to mimic the extracellular matrix (ECM), facilitating cell adhesion, proliferation, and differentiation [32].

Stem cell therapy, particularly involving MSCs and ADSCs, has shown promise in regenerative applications due to their multipotent differentiation potential and immunomodulatory properties [33,34]. MSCs can differentiate into osteogenic, chondrogenic, and adipogenic lineages, making them suitable for bone, cartilage, and soft tissue engineering [35,36]. Similarly, ADSCs have demonstrated efficacy in soft tissue regeneration, providing a readily available and minimally invasive source of stem cells [37].

Bioprinting technology has revolutionized tissue engineering by enabling the precise layer-by-layer deposition of cells and biomaterials, creating complex tissue structures that closely replicate native tissues [13,38]. This technology allows for the customization of scaffolds to match patient-specific anatomical and functional requirements, enhancing the integration and performance of engineered tissues [39].

Clinical Outcomes and Applications

The clinical applications of tissue engineering are vast, spanning orthopedics, cardiology, neurology, and plastic surgery. In orthopedics, tissue-engineered constructs for bone and cartilage repair have shown promising results in treating critical-sized bone defects and osteochondral injuries [26,40]. The use of MSCs and ADSCs in these applications has facilitated enhanced bone regeneration and cartilage repair, improving clinical outcomes [41,42].

In cardiology, the development of bioengineered vascular grafts and cardiac patches offers potential solutions for cardiovascular diseases, providing improved biocompatibility and reduced risk of graft failure compared to synthetic alternatives [43,44]. The integration of vascular endothelial growth factor (VEGF) and other angiogenic factors into these constructs has further enhanced their regenerative capacity, promoting neovascularization and tissue integration [45,46].

Neurological applications of tissue engineering focus on the regeneration of damaged neural tissues, leveraging the neurogenic potential of stem cells and bioactive scaffolds [47,48]. Constructs designed to support neural cell growth and differentiation have shown efficacy in promoting nerve regeneration and functional recovery in preclinical models [49,50].

In plastic and reconstructive surgery, tissue-engineered skin, fat, and muscle constructs offer innovative solutions for complex defects, improving both functional and aesthetic outcomes [51,52]. The use of ADSCs in these applications has been particularly effective, providing enhanced regenerative capacity and reducing donor site morbidity [5,6].

Challenges and Future Directions

Despite the significant advancements, the clinical translation of tissue engineering and regenerative medicine faces several challenges. Ensuring adequate vascularization of engineered constructs remains a critical issue, as it is essential for the survival and integration of the transplanted tissue [53,54]. Strategies such as prevascularization of scaffolds and the incorporation of angiogenic factors such as VEGF and fibroblast growth factor (FGF) are being explored to address this challenge [55,56].

Controlling the degradation rate of scaffolds to match the rate of tissue formation is another important consideration. Rapid degradation can compromise the structural integrity of the construct, while slow degradation can hinder tissue integration and function [57,58]. Advances in material science are focused on developing biodegradable polymers with tunable degradation rates to optimize scaffold performance [32,59].

Regulatory and cost barriers also limit the widespread clinical adoption of tissue-engineered products. The complex regulatory landscape for advanced therapies necessitates rigorous preclinical and clinical testing to ensure safety and efficacy [60,61]. Additionally, the high cost of production and the need for specialized facilities and expertise pose significant financial challenges [62,63]. Collaborative efforts between academia, industry, and regulatory bodies are essential to streamline the development and approval processes, reducing costs and accelerating clinical translation.

Limitations of the Current Review

While this systematic review provides comprehensive insights into the advantages of tissue engineering, it is important to acknowledge certain limitations. Firstly, the heterogeneity of study designs, patient populations, and outcome measures across the included studies may introduce variability that could affect the generalizability of the findings. Secondly, the majority of studies included in this review were conducted in controlled research settings, which may not fully reflect the complexities of real-world clinical practice. Lastly, the relatively short follow-up periods in some studies limit the ability to assess long-term outcomes and potential late complications associated with tissue-engineered constructs [23,25].

Moreover, the rapid pace of advancements in tissue engineering means that new technologies and methods may have emerged since the publication of the included studies. Continuous updates to the literature and systematic reviews are necessary to capture the latest developments and ensure that clinical practices evolve in line with emerging evidence [15,16].

Summary

The integration of tissue engineering and regenerative medicine represents a paradigm shift in reconstructive surgery, offering significant advantages over traditional techniques in terms of scar quality, postoperative pain, and patient satisfaction. This review highlights the superior clinical outcomes associated with tissue-engineered constructs, supported by extensive literature. However, challenges such as ensuring adequate vascularization, controlling scaffold degradation, and overcoming regulatory and cost barriers must be addressed to fully realize the potential of these innovative therapies. Continued research and development are essential to advance the field of tissue engineering and regenerative medicine, ultimately improving patient care and outcomes in reconstructive surgery.

## Conclusions

This systematic review highlights the significant advantages of tissue engineering over traditional reconstructive methods, particularly in scar quality, postoperative pain, and patient satisfaction. Across 14 studies, tissue-engineered constructs consistently delivered superior cosmetic outcomes, with 85-90% of patients reporting minimal scarring. Additionally, these techniques were associated with quicker pain resolution and significantly higher patient satisfaction, reaching 90-95% compared to 70-85% with conventional approaches.

The potential for tissue engineering to revolutionize reconstructive surgery is clear, with applications across various medical fields. However, challenges remain, including ensuring adequate vascularization, managing scaffold degradation, navigating regulatory frameworks, and addressing cost concerns. Despite these hurdles, ongoing advancements are likely to further enhance the scalability and clinical relevance of tissue engineering, paving the way for its broader adoption in patient care.
